# Distress disclosure and health behaviours in patients with stroke: a theory-informed serial mediation model of social support, self-efficacy, and self-management

**DOI:** 10.3389/fneur.2026.1896437

**Published:** 2026-08-21

**Authors:** Xiaoqin Yang, Yan Han, Yuntian Liu, Huaqing Fan, Jie Wang, Yan He, Feipeng Li

**Affiliations:** 1 Affiliated Baotou Clinical College of Inner Mongolia Medical University, Baotou, Inner Mongolia, China; 2 Department of Neurology, Baotou Central Hospital, Baotou, Inner Mongolia, China; 3 Department of Neurosurgery, Baotou Central Hospital, Baotou, Inner Mongolia, China; 4 Department of Nursing, Baotou Central Hospital, Baotou, Inner Mongolia, China

**Keywords:** distress disclosure, health behaviours, self-efficacy, self-management, serial mediation, social support, stroke

## Abstract

**Aim:**

To examine the association between distress disclosure and health behaviours among patients with stroke and to test whether social support, self-efficacy and self-management statistically mediate this association independently and in sequence.

**Methods:**

A convenience sample of 473 patients with stroke was recruited from the Department of Neurology, Baotou Central Hospital, Inner Mongolia, China, between August 2025 and March 2026. Participants completed a sociodemographic and clinical characteristics form, the Distress Disclosure Index, Social Support Rating Scale, Stroke Self-Efficacy Questionnaire, Stroke Self-Management Scale and Health Behaviour Scale for Stroke Patients. Descriptive statistics, Pearson correlations, hierarchical multiple linear regression and serial mediation analyses were conducted using IBM SPSS Statistics 29.0 and PROCESS macro version 5.0, Model 6. Age, stroke type, modified National Institutes of Health Stroke Scale (modified NIHSS) score and Barthel Index (BI) score were included as covariates.

**Results:**

The mean health-behaviour score was 60.65 (SD 12.32), indicating a moderate level. Distress disclosure was positively correlated with health behaviours (*r* = 0.828, *p* < 0.001) and remained a significant correlate after adjustment for covariates (B = 1.333, beta = 0.819, *p* < 0.001). The total effect of distress disclosure on health behaviours was 1.3327 (95% CI: 1.2494 to 1.4160), the direct effect was 0.2032 (95% CI: 0.0635 to 0.3429), and the total indirect effect was 1.1295 (95% CI: 0.9875 to 1.2884). Social support, self-efficacy and self-management each showed significant indirect statistical effects. The largest specific indirect effect was through social support alone (effect = 0.4749; 35.63% of the total effect). The full serial indirect association through social support, self-efficacy and self-management was also significant (effect = 0.0276; 95% CI: 0.0121 to 0.0479).

**Conclusion:**

Distress disclosure was strongly associated with health behaviours among patients with stroke, and this association was accompanied by significant indirect statistical associations via social support, self-efficacy and self-management. The findings support an expression-support-efficacy-management framework that may inform the design and prospective evaluation of communication-centred and self-management-oriented nursing interventions.

## 1 Introduction

Stroke remains one of the leading causes of death and disability worldwide and is characterised by high incidence, substantial long-term disability and an increasing disease burden. The Global Burden of Disease Study estimated that, in 2021, there were approximately 93.8 million prevalent cases and 11.9 million incident cases of stroke globally, and the burden of stroke continued to rise from 1990 to 2021 (1). The World Stroke Organization-Lancet Neurology Commission further warned that, without more effective prevention and management strategies, global deaths from stroke may increase substantially by 2050 (2). Beyond mortality and neurological impairment, stroke affects motor, cognitive, emotional and social functioning, creating sustained pressure on families, caregivers and healthcare systems (3, 4). In this context, secondary prevention after stroke is not simply a matter of pharmacological treatment or scheduled follow-up; it also depends on whether patients can maintain health-promoting behaviours throughout recovery and long-term disease management. The AHA/ASA secondary prevention guideline identifies blood pressure control, healthy diet, regular physical activity, smoking cessation and comprehensive risk-factor management as central components of recurrent stroke prevention, all of which require sustained and active behavioural engagement from patients (5).

Despite the centrality of health behaviours in secondary prevention, behavioural implementation among stroke survivors remains suboptimal. Recent reviews and population-based evidence indicate that risk-factor management and adherence to physical activity recommendations are insufficient in many stroke survivors (6, 7). These findings point to a persistent implementation gap in long-term stroke management: even when patients understand the importance of lifestyle modification and secondary prevention, they may struggle to sustain physical activity, dietary regulation, smoking and alcohol reduction, medication adherence and rehabilitation training because of physical limitations, emotional distress and social-contextual barriers. Scientific statements and reviews on psychosocial health after stroke further suggest that behavioural change is shaped by multiple interacting factors, including mood, stress, fatigue, adjustment and support resources (8, 9). Identifying modifiable psychosocial factors associated with sustained health behaviours is therefore clinically important.

Distress disclosure, a clinically relevant form of self-disclosure, may represent one such upstream psychosocial-behavioural factor. Self-disclosure broadly refers to the process through which individuals communicate inner feelings, thoughts, needs and concerns to others; in the context of illness, distress disclosure more specifically reflects patients’ willingness to share negative emotions, illness-related difficulties and support needs (10). Prior research suggests that self-disclosure is associated with trust, mutual understanding, relational intimacy and psychological well-being (11). For patients after stroke, functional limitations, role changes, fear of recurrence and emotional fluctuations are common (4, 12), and the AHA scientific statement on psychosocial health after stroke highlights the high prevalence of depression, anxiety, stress, fatigue and reduced quality of life in this population (9). Patients who are more willing to express illness experiences, rehabilitation difficulties and care needs may be better positioned to release emotional distress, clarify problems and mobilise support. Conversely, persistent inhibition of disclosure may be associated with greater psychological burden, avoidance and lower behavioural engagement.

Social support may serve as a key bridge between distress disclosure and health behaviours. Social support encompasses emotional, informational and instrumental assistance obtained from family members, friends, peers and healthcare professionals (13). Disclosure may make an individual’s lived situation and unmet needs more visible to others, which can be associated with greater responsiveness, support exchange and relational adjustment (11, 14). In stroke care, systematic reviews and randomised controlled trials have shown that interventions centred on peer support, supportive communication and nurse-led support can improve psychosocial outcomes among stroke survivors (15, 16). These findings indicate that support is not only an emotional resource but also a potentially important external condition for sustained rehabilitation engagement and behavioural adherence. Accordingly, higher distress disclosure may be associated with better health behaviours partly through greater access to, and perception of, social support.

Self-efficacy may be a more proximal psychological correlate linking social support to health behaviours. Self-efficacy refers to individuals’ confidence in their capacity to perform specific behaviours and cope with task-related demands, and it is a central construct in theories of health-behaviour initiation, persistence and maintenance (17). Randomised evidence from stroke self-management support programmes has shown improvements in self-efficacy and outcome expectations for self-management behaviours (18). Recent reviews have also identified self-efficacy as a core construct repeatedly targeted in stroke self-management interventions and as a mechanism through which self-management skills may be translated into action (17). Social support may therefore be associated with health behaviours not merely because help is available, but because supportive interactions may strengthen patients’ confidence in managing illness and engaging in rehabilitation. Greater self-efficacy may, in turn, be associated with better adherence to medication, dietary regulation, rehabilitation exercises and healthier lifestyles.

Building on this logic, self-management may represent the behavioural process through which self-efficacy is operationalised in daily life. Self-management refers to the active day-to-day process by which people with chronic conditions monitor disease status, manage symptoms, use medications appropriately, control risks and adjust behaviours. A systematic review in International Journal of Stroke noted that self-management interventions have been recommended in multiple international stroke guidelines to improve health-related outcomes after stroke, with self-efficacy widely regarded as a key construct underpinning such interventions (17). In the stroke context, self-management is not an abstract attitude; rather, it involves the practical routines through which knowing what should be done and believing that one can do it are reflected in concrete actions, such as medication adherence, regular routines, rehabilitation exercises, follow-up visits and risk-factor control.

The present study was guided by Social Cognitive Theory (19) and the Individual and Family Self-Management Theory (20). Social Cognitive Theory highlights self-efficacy as a central construct in behavioural initiation, persistence and maintenance. The Individual and Family Self-Management Theory provides a complementary framework in which social support can be conceptualised as a contextual resource, self-efficacy as a proximal psychological process, self-management as the behavioural process and health behaviours as the outcome of interest. Together, these theories support a hypothesised statistical sequence in which distress disclosure is positioned as a psychosocial communication factor: patients who disclose illness-related distress may report greater social support, stronger self-efficacy, more active self-management and healthier behaviours.

Although previous studies have separately examined risk-factor control, psychosocial adjustment, self-management support and supportive interventions after stroke, relatively little is known about the integrated association linking distress disclosure, social support, self-efficacy, self-management, and health behaviours. Much of the existing stroke literature has focused on the effectiveness of self-management support programmes, psychosocial interventions, transitional care or person-centred management. In the broader psychological literature, self-disclosure has often been used to explain relational well-being, psychological distress and social connectedness, but it has rarely been incorporated into models of health-behaviour formation among stroke survivors. Thus, existing evidence remains fragmented regarding whether disclosure-related processes are associated with external resources, internal beliefs and behavioural enactment in relation to health behaviours after stroke.

Therefore, this study aimed to examine the association between distress disclosure and health behaviours among patients with stroke and to test the independent and serial statistical mediating roles of social support, self-efficacy and self-management in this association. Based on the literature and theoretical framework described above, we proposed the following hypotheses: H1, distress disclosure would be positively associated with health behaviours; H2, social support would statistically mediate the association between distress disclosure and health behaviours; H3, self-efficacy would statistically mediate the association between distress disclosure and health behaviours; H4, self-management would statistically mediate the association between distress disclosure and health behaviours; and H5, social support, self-efficacy and self-management would jointly form a serial statistical mediation pathway between distress disclosure and health behaviours.

## 2 Methods

### 2.1 Study design and reporting guideline

This was a single-centre, cross-sectional observational study. The study was designed and reported in accordance with the Strengthening the Reporting of Observational Studies in Epidemiology (STROBE) statement (21). Because all core variables were measured at a single time point, the mediation model was treated as a theory-informed statistical model of indirect associations rather than evidence of temporal or causal ordering. This interpretation is consistent with methodological concerns that cross-sectional mediation analyses can yield biased estimates of longitudinal mediation processes (22).

### 2.2 Study setting and participants

A convenience sample of patients with stroke was recruited from the Department of Neurology, Baotou Central Hospital, Inner Mongolia, China, between August 2025 and March 2026.

Patients were eligible if they met all of the following criteria: (1) aged 18–80 years; (2) had a diagnosis of stroke confirmed on the basis of clinical manifestations and cranial computed tomography or magnetic resonance imaging findings; (3) had a Mini-Mental State Examination score of at least 24 and no severe psychiatric disorder that would substantially interfere with informed participation or questionnaire completion, such as schizophrenia or severe depressive disorder; (4) were conscious, had no evident cognitive impairment, had sufficient verbal communication ability, and were able to complete the questionnaire independently or with standardised assistance; and (5) provided informed consent and agreed to participate voluntarily.

Patients were excluded if they: (1) had a transient ischaemic attack; (2) had severe aphasia, cognitive impairment, psychiatric disorder, or severe hearing or visual impairment that precluded questionnaire completion independently or with assistance; (3) had severe cardiac, hepatic or renal failure, malignant tumours, or other severe physical illnesses; (4) were in an acute or critical condition and were unable to cooperate with the survey; or (5) refused to participate or withdrew during the study.

Patients with severe depressive disorder were excluded because the study required completion of a relatively lengthy self-report battery and stable comprehension, communication and sustained attention. This criterion was intended to improve the interpretability of questionnaire responses; however, it excluded a clinically important subgroup whose distress disclosure, social support, self-efficacy and health behaviours may differ from those of the included participants. The resulting limitation to generalisability is acknowledged in the Discussion.

The sample size was considered adequate based on empirical guidance for regression-based survey research and methodological recommendations for mediation analysis rather than as a definitive *a priori* causal-mediation power calculation. First, the present study included 43 observed variables, and a commonly used participant-to-variable rule for survey and regression-based analyses suggests that at least 10 participants per observed variable can be used as a lower-bound adequacy criterion, corresponding to 430 participants in this study (23). Second, simulation studies of mediation models indicate that larger samples are generally required to detect indirect effects with acceptable precision, especially when the indirect effect is small to moderate and when models include multiple mediators (24, 25). After allowing for an approximately 10% invalid-response rate, the target sample size was set at about 473. A total of 473 valid questionnaires were ultimately included in the final analysis.

### 2.3 Measures

Data were collected using a sociodemographic and clinical characteristics form and five validated instruments: the Distress Disclosure Index, the Social Support Rating Scale, the Stroke Self-Efficacy Questionnaire, the Stroke Self-Management Scale and the Health Behaviour Scale for Stroke Patients. In the main analyses, distress disclosure, social support, self-efficacy, self-management, and health behaviours were treated as continuous variables.

#### 2.3.1 Sociodemographic and clinical characteristics

A sociodemographic and clinical characteristics form was developed by the research team to collect participant-level information. Sociodemographic variables included age, sex, education level, marital status, employment status, living arrangement, place of residence, primary caregiver, monthly personal income, medical payment method, religious belief, personality and type of work. Clinical variables included perceived understanding of stroke, family history of stroke, stroke type, stroke severity, stroke duration, number of stroke episodes, limb dysfunction, self-care ability and number of chronic diseases. Stroke severity was assessed using the modified National Institutes of Health Stroke Scale (modified NIHSS), and self-care ability was assessed using the Barthel Index (BI). In this study, the modified NIHSS referred to the 11-item modified NIH Stroke Scale; total scores range from 0 to 31, with higher scores indicating more severe neurological impairment. For descriptive grouping, modified NIHSS scores were classified as mild stroke (0–4 points) or moderate stroke (5–15 points); no participants had scores above the moderate range. The BI ranges from 0 to 100, with higher scores indicating greater independence in activities of daily living. BI categories were defined as total dependence (0–20 points), severe dependence (21–60 points), moderate dependence (61–90 points), mild dependence (91–99 points) and independence (100 points). These variables were used to describe the sample and to identify potential covariates for subsequent analyses.

#### 2.3.2 Distress Disclosure Index

Distress disclosure was assessed using the Distress Disclosure Index (DDI) (10). The DDI is a unidimensional 12-item scale rated on a five-point Likert-type scale from 1 = strongly disagree to 5 = strongly agree. Items 1, 3, 6, 7, 11 and 12 are positively worded, whereas items 2, 4, 5, 8, 9 and 10 are reverse scored. Total scores range from 12 to 60, with higher scores indicating a greater tendency to disclose distress-related thoughts and feelings. Scores of 12–29, 30–44 and 45–60 indicate low, moderate and high levels of distress disclosure, respectively. Previous validation work reported good reliability, with a Cronbach’s alpha of 0.866, split-half reliability of 0.847 and test–retest reliability of 0.780.

#### 2.3.3 Social Support Rating Scale

Social support was measured using the Social Support Rating Scale (SSRS). The SSRS contains 10 items across three dimensions: objective support, subjective support and support utilisation. Objective support, subjective support and support utilisation are measured by three, four and three items, respectively. Items are scored using four-point or weighted scoring methods according to item format. Total scores range from 12 to 66, with higher scores indicating greater perceived and available social support. Total scores of 22 or lower, 23–44 and 45 or higher are generally interpreted as low, moderate and high levels of social support, respectively. Previous studies reported good reliability, with a Cronbach’s alpha of 0.89 and test–retest reliability of 0.92.

#### 2.3.4 Stroke Self-Efficacy Questionnaire

Stroke-related self-efficacy was assessed using the Stroke Self-Efficacy Questionnaire (SSEQ), a stroke-specific instrument designed to assess confidence in performing daily and self-management tasks after stroke. It contains 11 items across two dimensions: daily activity self-efficacy, comprising six items, and self-management self-efficacy, comprising five items. Each item is scored from 1 = not at all confident to 10 = completely confident. Total scores range from 11 to 110, with higher scores indicating stronger stroke-related self-efficacy. Previous validation of the Chinese version reported excellent internal consistency, with a Cronbach’s alpha of 0.969.

#### 2.3.5 Stroke Self-Management Scale

Self-management was measured using the Stroke Self-Management Scale (SSMS), which contains 50 items in seven domains: disease management, medication safety management, dietary management, daily-life management, emotional management, social functioning and interpersonal relationship management, and rehabilitation exercise management. Items are rated on a five-point Likert-type scale, with higher scores indicating better self-management. The total score can be converted into a score index using the following formula: actual total score divided by the theoretical maximum score multiplied by 100%. A score index below 60, 60–80% and above 80% indicates poor, moderate and good self-management, respectively. Previous validation reported good internal consistency, with a Cronbach’s alpha of 0.835.

#### 2.3.6 Health Behaviour Scale for Stroke Patients

Health behaviours were measured using the Health Behaviour Scale for Stroke Patients (HBS-SP). The HBS-SP contains 25 items covering exercise, nutrition, monitoring responsibility, smoking-related behaviours, alcohol-related behaviours and medication-related behaviours. Each item is rated on a four-point Likert-type scale from 1 = never to 4 = routinely. Total scores range from 25 to 100, and mean item scores range from 1 to 4. Higher scores indicate better health behaviours. The original scale showed good internal consistency, with a Cronbach’s alpha of 0.88.

#### 2.3.7 Conceptual distinction between self-management and health behaviours

Although the SSMS and HBS-SP both include behaviour-related content, they were treated as conceptually distinct constructs in this study. The SSMS was used to capture patients’ broader self-regulatory capacity and routines for managing stroke-related illness, including disease management, medication safety, dietary management, daily-life management, emotional management, social functioning and rehabilitation exercise management. In contrast, the HBS-SP was used as the outcome measure and focuses on the reported performance of health-promoting and secondary-prevention behaviours, including exercise, nutrition, monitoring responsibility, smoking-related behaviours, alcohol-related behaviours and medication-related behaviours. Thus, self-management was conceptualised as a broader management process, whereas health behaviours were conceptualised as specific behavioural performance. However, this distinction is theoretical rather than complete at the domain level: the two instruments share or closely approximate content related to medication, diet, daily-life or monitoring activities, and rehabilitation or exercise. Consequently, estimates involving the SSMS and HBS-SP may reflect both the proposed process-outcome relationship and shared behavioural content. These findings were therefore interpreted cautiously as statistical associations, with measurement redundancy and potential inflation of associations considered explicitly in the Discussion.

### 2.4 Data collection and quality control

Before data collection, the principal researcher provided standardised training to all data collectors regarding the study objectives, questionnaire content, administration procedures, completion requirements and precautions to ensure consistency across the survey process. During formal data collection, trained data collectors explained the study purpose, significance and questionnaire completion procedures to eligible patients. After informed consent had been obtained, paper questionnaires were distributed and collected onsite. Participants who were able to complete the questionnaire independently completed it according to their own circumstances. For participants with low literacy, writing difficulties or disease-related limitations but preserved comprehension and communication ability, trained data collectors read each item verbatim using a standardised script and recorded responses strictly according to the participants’ answers, thereby minimising information bias and interviewer-induced variation. After collection, completed questionnaires were checked for completeness. Two researchers independently organised and entered the data, cross-checked the entries against the original questionnaires and corrected discrepancies promptly to ensure data accuracy and completeness.

### 2.5 Statistical analysis

Data were analysed using IBM SPSS Statistics version 29.0 (IBM Corp., Armonk, NY, USA). Serial mediation analyses were conducted using the PROCESS macro for SPSS, version 5.0, Model 6. All statistical tests were two-sided, and a *p* value of <0.05 was considered statistically significant. Because the study was cross-sectional and all core variables were self-reported, mediation parameters were interpreted as indirect statistical associations rather than causal effects.

Before analysis, all data were checked for completeness and distributional characteristics. Categorical variables were summarised as frequencies and percentages. Continuous variables, including distress disclosure, social support, self-efficacy, self-management, and health behaviours, were summarised as means and standard deviations when approximately normally distributed, and as medians and interquartile ranges when distributions were skewed.

Differences in health-behaviour scores across sociodemographic and clinical characteristics were examined using independent-samples *t* tests or one-way analysis of variance, as appropriate. For variables that did not meet parametric assumptions, Mann–Whitney U tests or Kruskal-Wallis tests were used.

Pairwise associations among distress disclosure, social support, self-efficacy, self-management, and health behaviours were examined using Pearson correlation coefficients. Hierarchical multiple linear regression was then used to examine the association between distress disclosure and health behaviours after adjustment for covariates. Multicollinearity was assessed using variance inflation factors. Because all core variables were measured using self-report questionnaires at the same time point, common method variance and socially desirable responding were considered possible threats to interpretation (26). Potential common method variance was examined using Harman’s single-factor test by entering all core scale items into an unrotated exploratory factor analysis. The test was used as a preliminary diagnostic only. Because Harman’s test has limited sensitivity and cannot rule out common method variance, its result was not interpreted as definitive; interpretation also considered the same-source cross-sectional design, the magnitude of intercorrelations and potential construct overlap.

Covariates were selected on the basis of prior literature, theoretical considerations and clinical relevance to health-behaviour formation after stroke rather than by applying a purely data-driven significance threshold. Age was included as a demographic factor; stroke type and modified NIHSS score represented disease characteristics and neurological severity; and BI score represented functional independence. Univariate analyses were used to describe subgroup differences and inform interpretation, but statistical significance in these analyses was not treated as the sole criterion for covariate inclusion. Age, stroke type, modified NIHSS score and BI score were included as covariates in the hierarchical regression and serial mediation models to reduce potential confounding. Categorical covariates were dummy-coded before entry into the models.

In the serial mediation model, distress disclosure was specified as the independent variable, health behaviours as the dependent variable, and social support, self-efficacy and self-management as serial mediators. The mediators were entered in the following theory-informed order: social support, self-efficacy and self-management. Direct, indirect and total statistical effects were estimated. Indirect effects were tested using a non-parametric bootstrapping procedure with 5,000 resamples, an approach recommended for estimating confidence intervals for indirect effects (27). An indirect effect was considered statistically significant when the 95% bootstrap confidence interval did not include zero. Unstandardised coefficients were reported for regression and mediation analyses, whereas standardised coefficients were used in the path diagram.

### 2.6 Ethical considerations

The study protocol was reviewed and approved by the Ethics Committee of Baotou Central Hospital, Inner Mongolia, China (approval No. 2025-YJS-LS-078). Before questionnaire administration, all participants and/or their legal guardians were informed of the study purpose, voluntary nature of participation, confidentiality of data and their right to withdraw at any time. Informed consent was obtained from all participants and/or their legal guardians before participation.

## 3 Results

### 3.1 Participant characteristics and univariate analysis of health behaviours

A total of 473 patients with stroke were included in the analysis. The mean age was 55.17 years (SD 10.61). Patients aged 36–59 years accounted for the largest proportion of the sample (64.06%), followed by those aged 60 years or older (33.19%). Of the participants, 253 were men (53.49%) and 220 were women (46.51%). Regarding stroke type, 263 patients had ischaemic stroke (55.60%) and 210 had haemorrhagic stroke (44.40%). The mean modified NIHSS score was 5.84 (SD 1.80); 122 patients (25.79%) were classified as having mild stroke and 351 (74.21%) as having moderate stroke. The mean BI score was 69.20 (SD 12.34), and most participants had moderate dependence (*n* = 323, 68.29%). The mean total health-behaviour score was 60.65 (SD 12.32). Core sociodemographic and clinical variables are shown in Table 1, and additional descriptive variables are presented in Supplementary Table 1.

**Table 1:** Core participant characteristics and univariate analysis of health behaviours.

Characteristic	*n*	% or mean ± SD	Health-behaviour score	*t*/F	*p*
Age, years		55.17 ± 10.61			
≤35	13	2.75	61.38 ± 16.11	3.089	0.046*
36–59	303	64.06	61.65 ± 12.13	3.089	0.046*
≥60	157	33.19	58.66 ± 12.21	3.089	0.046*
Sex					
Male	253	53.49	61.23 ± 11.68	1.094	0.274
Female	220	46.51	59.99 ± 13.02	1.094	0.274
Education level					
Primary school or below	114	24.10	62.26 ± 12.43	2.785	0.026*
Junior high school	200	42.28	58.46 ± 11.66	2.785	0.026*
Senior high school/technical secondary school	77	16.28	62.22 ± 12.02	2.785	0.026*
College diploma	40	8.46	62.48 ± 12.29	2.785	0.026*
Bachelor degree or above	42	8.88	62.10 ± 14.54	2.785	0.026*
Monthly personal income, CNY					
<3,000	235	49.68	59.92 ± 11.43	4.117	0.017*
3,000–6,000	156	32.98	62.85 ± 12.22	4.117	0.017*
>6,000	82	17.34	58.56 ± 14.38	4.117	0.017*
Medical payment method					
Urban resident basic medical insurance	180	38.06	59.09 ± 11.91	2.869	0.036*
Urban employee basic medical insurance	194	41.01	61.80 ± 13.14	2.869	0.036*
New rural cooperative medical scheme	88	18.60	60.39 ± 11.27	2.869	0.036*
Self-pay or other	11	2.33	68.00 ± 8.15	2.869	0.036*
Perceived understanding of stroke					
No understanding	102	21.57	57.68 ± 12.62	6.124	0.002*
Partial understanding	300	63.42	60.79 ± 12.32	6.124	0.002*
Full understanding	71	15.01	64.24 ± 10.98	6.124	0.002*
Stroke type					
Haemorrhagic	210	44.40	59.22 ± 12.83	−2.260	0.024*
Ischaemic	263	55.60	61.79 ± 11.81	−2.260	0.024*
Stroke severity (modified NIHSS score)		5.84 ± 1.80			
Mild stroke	122	25.79	62.56 ± 11.03	1.989	0.047*
Moderate stroke	351	74.21	59.99 ± 12.69	1.989	0.047*
Stroke duration					
<1 month	316	66.81	60.33 ± 12.26	2.118	0.097
1–3 months	104	21.99	60.50 ± 12.61	2.118	0.097
3 months + 1 day to 6 months	43	9.10	60.16 ± 12.06	2.118	0.097
6 months + 1 day to 1 year	10	2.10	70.20 ± 10.17	2.118	0.097
Barthel Index (BI) score		69.20 ± 12.34			
Severe dependence	141	29.81	60.04 ± 12.96	3.596	0.028*
Moderate dependence	323	68.29	60.62 ± 12.03	3.596	0.028*
Mild dependence	9	1.90	71.33 ± 7.94	3.596	0.028*

Values are *n* (%), mean ± SD, or health-behaviour score mean ± SD. Stroke severity categories were mild stroke (modified NIHSS 0–4 points) and moderate stroke (modified NIHSS 5–15 points). BI categories shown were severe dependence (21–60 points), moderate dependence (61–90 points) and mild dependence (91–99 points). Non-core sociodemographic and clinical variables are shown in Supplementary Table 1. *p ≤ 0.05.

Univariate analyses showed that health-behaviour scores differed significantly by age, education level, monthly personal income, medical payment method, perceived understanding of stroke, stroke type, modified NIHSS-defined stroke severity and BI-defined self-care ability level (all *p* < 0.05). Specifically, health-behaviour scores were higher among patients with ischaemic stroke than among those with haemorrhagic stroke (61.79 [SD 11.81] vs. 59.22 [SD 12.83], *t* = −2.260, *p* = 0.024), and higher among patients with mild stroke than among those with moderate stroke (62.56 [SD 11.03] vs. 59.99 [SD 12.69], *t* = 1.989, *p* = 0.047). For age, education level, monthly personal income, medical payment method, perceived understanding of stroke and BI-defined self-care ability level, overall between-group differences were statistically significant (all *p* < 0.05; Table 1).

Additional variables, including marital status, employment status, living arrangement, place of residence, primary caregiver, religious belief, personality type, type of work, smoking status, alcohol use, family history of stroke, number of stroke episodes, limb dysfunction and number of chronic diseases, are summarised in Supplementary Table 1. These variables were not retained in the main text table because they were not central to the adjusted models.

### 3.2 Scores and correlations among distress disclosure, social support, self-efficacy, self-management, and health behaviours

Among the 473 patients with stroke, the mean scores were 60.65 (SD 12.32) for health behaviours, 43.70 (SD 8.60) for social support, 73.18 (SD 15.93) for self-efficacy, 155.85 (SD 33.88) for self-management and 46.12 (SD 7.58) for distress disclosure. Harman’s single-factor test showed that the first unrotated factor accounted for 36% of the total variance, which was below the commonly used threshold of 40%; however, this result does not exclude common method variance. Given the same-source self-report design and the magnitude of several intercorrelations, the associations were interpreted cautiously. Pearson correlation analysis showed that health behaviours were positively correlated with social support, self-efficacy, self-management and distress disclosure, with correlation coefficients of 0.841, 0.788, 0.810 and 0.828, respectively (all *p* < 0.001). Significant positive correlations were also observed between social support and self-efficacy (*r* = 0.738), self-management (*r* = 0.719) and distress disclosure (*r* = 0.859); between self-efficacy and self-management (*r* = 0.691) and distress disclosure (*r* = 0.722); and between self-management and distress disclosure (*r* = 0.757) (all *p* < 0.001; Table 2).

**Table 2:** Scores and correlations among distress disclosure, social support, self-efficacy, self-management, and health behaviours.

Variable	Score	Health behaviours	Social support	Self-efficacy	Self-management	Distress disclosure
Health behaviours	60.65 ± 12.32	1.000	--	--	--	--
Social support	43.70 ± 8.60	0.841***	1.000	--	--	--
Self-efficacy	73.18 ± 15.93	0.788***	0.738***	1.000	--	--
Self-management	155.85 ± 33.88	0.810***	0.719***	0.691***	1.000	--
Distress disclosure	46.12 ± 7.58	0.828***	0.859***	0.722***	0.757***	1.000

***p < 0.001. Dashes indicate not applicable.

### 3.3 Hierarchical multiple linear regression analysis of health behaviours

Hierarchical multiple linear regression was conducted with health-behaviour score as the dependent variable. Model 1 included age, stroke type, modified NIHSS score and BI score. This model was statistically significant (R2 = 0.043, adjusted R2 = 0.035, *F* = 5.233, *p* < 0.001), explaining 4.3% of the variance in health behaviours. After distress disclosure was added in Model 2, the model remained statistically significant (R2 = 0.693, adjusted R2 = 0.690, *F* = 210.759, *p* < 0.001), and the explained variance increased substantially compared with Model 1 (Delta R2 = 0.650, *p* < 0.001).

In the final model, age was negatively associated with health behaviours (B = −0.104, beta = −0.090, *t* = −3.291, *p* = 0.001, 95% CI: −0.166 to −0.042), whereas distress disclosure was positively associated with health behaviours (B = 1.333, beta = 0.819, *t* = 31.443, *p* < 0.001, 95% CI: 1.249 to 1.416). Stroke type, modified NIHSS score and BI score were not significantly associated with health behaviours after distress disclosure was entered into the model (all *p* > 0.05). Variance inflation factors were close to 1, indicating no evidence of serious multicollinearity (Table 3).

**Table 3:** Hierarchical multiple linear regression analysis of factors associated with health behaviours.

Variable	B	SE	beta	*t*	*p*	95% CI
Constant	3.986	3.148		1.266	0.206	−2.201 to 10.172
Age	−0.104	0.032	−0.090	−3.291	0.001	−0.166 to −0.042
Stroke type	0.227	0.641	0.009	0.354	0.724	−1.033 to 1.487
Modified NIHSS score	−0.052	0.177	−0.008	−0.295	0.768	−0.399 to 0.295
BI score	0.016	0.027	0.016	0.601	0.548	−0.037 to 0.069
Distress disclosure	1.333	0.042	0.819	31.443	<0.001	1.249 to 1.416

Dependent variable: health-behaviour score. Model 1 included age, stroke type, modified NIHSS score and BI score (R2 = 0.043, adjusted R2 = 0.035, F = 5.233, *p* < 0.001). Model 2 additionally included distress disclosure (R2 = 0.693, adjusted R2 = 0.690, F = 210.759, *p* < 0.001; Delta R2 = 0.650, Delta F = 988.688, *p* < 0.001). B, unstandardised coefficient; beta, standardised coefficient; CI, confidence interval.

### 3.4 Serial mediation analysis after adjustment for covariates

After adjustment for age, stroke type, modified NIHSS score and BI score, serial mediation analysis was performed using PROCESS macro version 5.0, Model 6. Distress disclosure was specified as the independent variable, health behaviours as the dependent variable, and social support, self-efficacy and self-management as serial mediators.

The regression model with social support as the dependent variable was statistically significant (R2 = 0.7426, *F* = 269.5120, *p* < 0.001), and distress disclosure was positively associated with social support (B = 0.9654, *p* < 0.001). The model with self-efficacy as the dependent variable was also significant (R2 = 0.6200, *F* = 126.7270, *p* < 0.001); both distress disclosure (B = 1.1150, *p* < 0.001) and social support (B = 0.5156, *p* < 0.001) were positively associated with self-efficacy. The model with self-management as the dependent variable was significant (R2 = 0.6122, *F* = 104.8876, *p* < 0.001); distress disclosure (B = 1.8313, *p* < 0.001), social support (B = 0.7841, *p* = 0.0007) and self-efficacy (B = 0.4817, *p* < 0.001) were positively associated with self-management. The model with health behaviours as the dependent variable was also significant (R2 = 0.8235, *F* = 270.5932, *p* < 0.001); distress disclosure (B = 0.2032, *p* = 0.0044), social support (B = 0.4920, *p* < 0.001), self-efficacy (B = 0.1656, *p* < 0.001) and self-management (B = 0.1152, *p* < 0.001) were positively associated with health behaviours.

The total effect of distress disclosure on health behaviours was 1.3327 (SE = 0.0424, 95% CI: 1.2494 to 1.4160, *p* < 0.001). After social support, self-efficacy and self-management were included in the model, the direct effect remained statistically significant (B = 0.2032, SE = 0.0711, 95% CI: 0.0635 to 0.3429, *p* = 0.0044). Bootstrapping with 5,000 resamples showed a significant total indirect effect of 1.1295 (BootSE = 0.0759, 95% CI: 0.9875 to 1.2884), as the confidence interval did not include zero.

**Figure 1:**
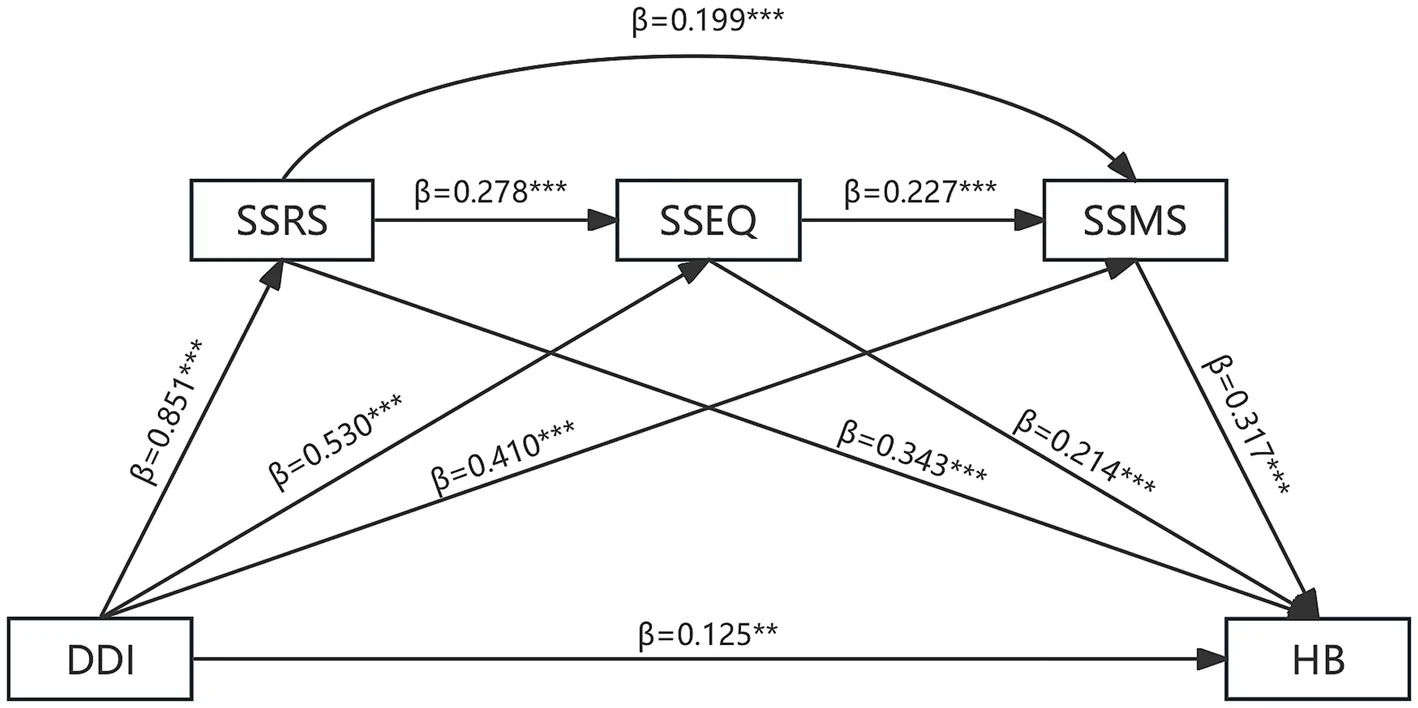
Theory-informed serial mediation model linking distress disclosure and health behaviours after adjustment for covariates. Values are standardised path coefficients (*beta*). Solid lines indicate statistically significant paths. DDI, distress disclosure; SSRS, social support; SSEQ, self-efficacy; SSMS, self-management; HB, health behaviours. Covariates included age, stroke type, modified NIHSS score and BI score. **p < 0.01, ***p < 0.001.

Decomposition of indirect effects showed that distress disclosure was indirectly associated with health behaviours through social support alone (Ind1 = 0.4749, BootSE = 0.0670, 95% CI: 0.3375 to 0.6007), self-efficacy alone (Ind2 = 0.1846, BootSE = 0.0507, 95% CI: 0.1033 to 0.3028) and self-management alone (Ind3 = 0.2109, BootSE = 0.0532, 95% CI: 0.1187 to 0.3266). Significant indirect effects were also observed through the sequential pathways of social support to self-efficacy (Ind4 = 0.0824, BootSE = 0.0300, 95% CI: 0.0364 to 0.1525), social support to self-management (Ind5 = 0.0872, BootSE = 0.0355, 95% CI: 0.0270 to 0.1646), self-efficacy to self-management (Ind6 = 0.0619, BootSE = 0.0185, 95% CI: 0.0297 to 0.1015), and social support to self-efficacy to self-management (Ind7 = 0.0276, BootSE = 0.0093, 95% CI: 0.0121 to 0.0479). These findings indicated that social support, self-efficacy and self-management each showed significant indirect statistical associations in the link between distress disclosure and health behaviours, and that the hypothesised serial indirect association was supported after adjustment for covariates (Table 4; Figure 1).

**Table 4:** Direct and indirect statistical effects in the serial mediation model after adjustment for covariates.

Effect path	Effect	BootSE/SE	95% CI	Proportion of total effect (%)
Total effect X → Y	1.3327	0.0424	1.2494 to 1.4160	100.00
Direct effect X → Y	0.2032	0.0711	0.0635 to 0.3429	15.25
Total indirect effect	1.1295	0.0759	0.9875 to 1.2884	84.75
Ind1 X → M1 → Y	0.4749	0.0670	0.3375 to 0.6007	35.63
Ind2 X → M2 → Y	0.1846	0.0507	0.1033 to 0.3028	13.85
Ind3 X → M3 → Y	0.2109	0.0532	0.1187 to 0.3266	15.83
Ind4 X → M1 → M2 → Y	0.0824	0.0300	0.0364 to 0.1525	6.18
Ind5 X → M1 → M3 → Y	0.0872	0.0355	0.0270 to 0.1646	6.54
Ind6 X → M2 → M3 → Y	0.0619	0.0185	0.0297 to 0.1015	4.64
Ind7 X → M1 → M2 → M3 → Y	0.0276	0.0093	0.0121 to 0.0479	2.07

X, distress disclosure; M1, social support; M2, self-efficacy; M3, self-management; Y, health behaviours. Covariates included age, stroke type, modified NIHSS score and BI score. SE is reported for total and direct effects; BootSE is reported for indirect effects. An indirect effect was considered significant when the bootstrap 95% CI did not include zero. Proportion of total effect (%) = effect/total effect x 100%.

## 4 Discussion

The principal contribution of this study is the integration of illness-related communication with contextual, cognitive and behavioural correlates of post-stroke health behaviour. Previous stroke research has more commonly examined social support, self-efficacy or self-management as separate determinants or intervention targets (17, 28), whereas distress disclosure has rarely been incorporated into an integrated health-behaviour model. The observed pattern is consistent with the proposition that patients’ willingness to express distress may be related to the visibility of their needs, perceived availability of support, confidence in performing rehabilitation tasks and the organisation of daily management routines. However, because all variables were measured concurrently, the model should be understood as a theory-informed representation of covarying processes rather than a verified temporal or causal sequence.

The mean health-behaviour score was 60.65 (SD 12.32), indicating that substantial scope remains for improving secondary-prevention and rehabilitation behaviours. This result is consistent with evidence that stroke survivors commonly experience barriers to sustained physical activity, medication adherence, dietary regulation and risk-factor monitoring (29, 30). It also reinforces the broader view that post-stroke care extends beyond acute treatment and depends on continuous, family-supported and context-sensitive behavioural management (3–5). Functional limitations, fatigue, emotional distress and role changes may increase the burden of behavioural engagement, while insufficient information, support or feedback may contribute to declining adherence after discharge (31).

The mean age of 55.17 years was comparatively young relative to many stroke cohorts. This may partly reflect the recruitment profile of this single-centre neurology sample, the eligibility range of 18–80 years and the requirement for preserved cognition, communication and ability to complete self-report questionnaires. These criteria may have reduced the representation of older patients with severe neurological, cognitive or communication impairment. The age distribution should therefore be considered when assessing the generalisability of the findings.

Distress disclosure remained strongly associated with health behaviours after adjustment for selected covariates. This finding extends post-stroke psychosocial research by identifying illness-related expression as a potentially informative communication correlate of behavioural engagement, rather than treating communication solely as a means of delivering education. The association should not be interpreted as evidence that disclosure causes healthier behaviour. One plausible interpretation is that patients who express worries, rehabilitation difficulties and unmet needs make those barriers more visible to family members and healthcare professionals, whereas patients who rarely disclose distress may have needs that remain hidden. Alternative and reciprocal explanations are also possible and are considered below.

As a potential direction for future practice and intervention development, communication assessment could extend beyond asking whether patients understand discharge instructions (9, 32). Nurses could consider brief prompts such as, ‘What worries you most about going home?’, ‘Which rehabilitation task is hardest to complete?’, ‘What prevents you from taking medicine or exercising as planned?’ and ‘Who can help you when these difficulties occur?’ Responses could be documented as candidate barriers to self-management and used to tailor education, referral and follow-up plans. These prompts and associated communication strategies were not evaluated in the present study and should therefore be regarded as components for future prospective testing rather than evidence-based effects demonstrated by the current data.

The indirect association through social support alone accounted for the largest proportion of the total effect in this sample, whereas the complete serial pathway accounted for a much smaller proportion. This statistical ranking does not establish that social support is the most important causal mechanism or that interventions targeting support would produce the greatest improvement in health behaviours. Rather, it identifies social support as a plausible component for future prospective and intervention research. This interpretation is clinically relevant because post-stroke behaviours may be facilitated by family reminders, transportation and meal assistance, medication supervision, rehabilitation encouragement, peer modelling and professional feedback (33), but the comparative effectiveness of support-focused strategies cannot be inferred from the present cross-sectional model.

Potential strategies for future testing include constructing a support map before discharge, identifying the main and backup caregivers, linking patients to peer or community resources, and clarifying professional contact points for problems. Family caregivers could be trained in specific supportive tasks, such as medication reminders, preparation of low-salt meals, encouragement of safe activity and support for follow-up attendance. Peer support and follow-up calls or digital messages may also be considered for patients with limited family support or low confidence (34). These approaches are practice implications derived from the observed associations and require evaluation in prospective studies.

Self-efficacy and self-management were also significant indirect correlates in the model. These findings are consistent with stroke self-management evidence showing that self-management interventions often target confidence, problem-solving, goal setting and behavioural routines (17, 28, 35, 36). From a nursing perspective, support is more likely to be useful when it is converted into patients’ confidence that they can perform specific behaviours and into concrete daily routines.

Potential components for future intervention development include graded task design, short-cycle goal setting, performance feedback and problem-solving (37). For example, rather than advising patients to ‘exercise more’, a nurse could help define a safe walking or limb-training target, identify warning signs, specify when and where the activity will occur, and review progress at follow-up. Medication, diet, monitoring and rehabilitation behaviours could be incorporated into a one-page self-management plan with checkboxes or a diary. These strategies are consistent with the model and previous intervention literature, but their effectiveness was not tested in the present study.

The full serial pathway from distress disclosure to social support, self-efficacy, self-management, and health behaviours was statistically significant, but its proportion of the total effect was small. Although this finding supports the theoretical plausibility of the proposed expression-support-efficacy-management sequence, the results mainly suggest a broader network of indirect statistical associations rather than a dominant stepwise chain. Therefore, the model should be presented as a theory-informed pattern of indirect associations rather than as proof that every patient moves through these stages in a fixed causal order. The strongest practical implication is not that the complete sequence represents the only mechanism, but that communication, support, confidence and self-management routines can be assessed together when designing post-stroke nursing care.

The exceptionally high correlations among the core variables require cautious interpretation. Correlations of 0.81–0.84 between health behaviours and distress disclosure, social support and self-management, together with the correlation of 0.86 between distress disclosure and social support, are unusually high for distinct psychosocial self-report constructs. These magnitudes may reflect genuine clustering of psychosocial resources and behavioural engagement, but they may also reflect common method variance, socially desirable or acquiescent response styles, construct overlap and measurement redundancy. The conceptual proximity between the SSMS and HBS-SP is particularly important: although the SSMS was intended to represent a broad management process and the HBS-SP to represent behavioural performance, both include medication-, diet-, monitoring-, daily-life- and rehabilitation-related content. Accordingly, the regression and indirect-effect estimates may partly capture shared item or domain content rather than fully distinct processes, and their magnitude may be inflated. Harman’s single-factor result does not resolve this concern. Future studies should evaluate discriminant validity, conduct dimension-level sensitivity analyses or latent-variable modelling, use longitudinal or multi-informant designs, and incorporate objective indicators such as blood pressure logs, step counts, pharmacy refill data, rehabilitation attendance and follow-up completion.

One possible nursing workflow for future evaluation could involve five linked steps: screening for disclosure difficulty; translating expressed concerns into an individualised barrier list; activating family, peer and healthcare support; using goal setting, teach-back, modelling and feedback to strengthen confidence for specific behaviours; and translating goals into a written self-management plan covering medication, diet, physical activity, symptom monitoring, risk-factor control and follow-up. This workflow illustrates how the expression-support-efficacy-management framework might be operationalised, but it was not tested in the current study and requires prospective feasibility and effectiveness evaluation.

This study has several strengths. It developed and tested a theory-informed serial mediation model linking distress disclosure to health behaviours among patients with stroke. By examining social support, self-efficacy and self-management simultaneously, the study extends previous work that has often focused on single psychosocial or behavioural factors and provides a more integrated description of factors associated with health behaviours after stroke. The sample size was considered adequate for the planned regression- and mediation-based analyses, and the model was adjusted for selected demographic and clinical covariates.

Several limitations should also be acknowledged. First, the cross-sectional design precludes causal inference and limits the ability to establish temporal ordering among distress disclosure, social support, self-efficacy, self-management, and health behaviours. The proposed sequence was theoretically specified rather than empirically verified; reciprocal or alternative pathways cannot be excluded. Reverse causation is plausible: patients who already engage in healthier behaviours may participate more consistently in rehabilitation, have more contact with healthcare professionals, receive or perceive greater social support, develop stronger self-efficacy and become more willing to disclose distress. The serial mediation model should therefore be interpreted as a theory-informed statistical pathway rather than evidence of causality. Second, participants were recruited from a single centre using convenience sampling, which may limit generalisability. The relatively young mean age, the absence of patients with modified NIHSS scores above the moderate range, and the requirement for sufficient cognition and communication mean that the findings may be most applicable to younger patients with mild-to-moderate stroke who can complete self-report questionnaires. In addition, excluding patients with severe depressive disorder omitted a clinically important subgroup whose disclosure, support, efficacy and health behaviours may differ; the findings should not be extrapolated to that group. Third, the same-source self-report design may have introduced reporting bias, common method variance and socially desirable or acquiescent responding. Harman’s single-factor test was only a preliminary diagnostic and cannot rule out these influences, particularly given several correlations above 0.80. Fourth, although the SSMS and HBS-SP were conceptually distinguished, their overlapping medication-, diet-, daily-life-, monitoring- and rehabilitation-related content may have inflated associations involving self-management and health behaviours and may limit interpretation of the mediator and outcome as fully distinct constructs. Fifth, although age, stroke type, modified NIHSS score and BI score were adjusted for, other potentially relevant factors, such as depression severity, family functioning, socioeconomic stress and disease duration, were not included in the final model. Future multicentre longitudinal, multi-method and intervention studies are needed to evaluate temporal ordering, discriminant validity and whether disclosure-oriented or support-oriented strategies improve long-term health behaviours.

## 5 Conclusion

In this cross-sectional study of patients with stroke, health behaviours were at a moderate level. Distress disclosure was positively associated with health behaviours, and social support, self-efficacy and self-management showed significant independent and serial indirect statistical associations in this relationship. The findings may inform the future development and prospective evaluation of nursing strategies addressing illness-related expression, social support, self-efficacy and self-management routines. Because of the cross-sectional design, the possibility of reverse causation, same-source self-report measurement, exceptionally high intercorrelations and partial construct overlap between self-management and health-behaviour measures, these results should be interpreted as evidence of associations rather than causality or comparative intervention effectiveness.

## Data Availability

The raw data supporting the conclusions of this article will be made available by the authors, without undue reservation.
