# Supplementary material for: Distress disclosure and health behaviours in patients with stroke: a theory-informed serial mediation model of social support, self-efficacy, and self-management
Source: Front Neurol. 2026 Aug 21;17:1896437. doi: 10.3389/fneur.2026.1896437 (PMC13541430; doi:10.3389/fneur.2026.1896437)
Supplement: Supplementary file 1 [file Table_1.DOCX]

Supplementary Table 1 Additional participant characteristics and univariate analysis of health behaviours.

| Characteristic | n | % or mean ± SD | Health-behaviour score | t/F | P |
| --- | --- | --- | --- | --- | --- |
| Marital status |  |  |  |  |  |
| Married | 447 | 94.50 | 60.69±11.00 | 0.224 | 0.825 |
| Unmarried/divorced/widowed | 26 | 5.50 | 59.92±17.30 | 0.224 | 0.825 |
| Employment status |  |  |  |  |  |
| Employed | 198 | 41.86 | 61.60±13.24 | 2.469 | 0.061 |
| Retired | 174 | 36.79 | 59.16±11.77 | 2.469 | 0.061 |
| Farmer | 92 | 19.45 | 60.67±11.22 | 2.469 | 0.061 |
| Unemployed | 9 | 1.90 | 68.44±8.82 | 2.469 | 0.061 |
| Living arrangement |  |  |  |  |  |
| Living alone | 17 | 3.59 | 62.71±9.03 | 0.295 | 0.745 |
| Living with family | 453 | 95.77 | 60.52±12.44 | 0.295 | 0.745 |
| Living in nursing institution | 3 | 0.64 | 58.67±12.06 | 0.295 | 0.745 |
| Place of residence |  |  |  |  |  |
| Rural | 91 | 19.24 | 61.08±11.21 | 1.505 | 0.223 |
| Town | 327 | 69.13 | 60.99±12.03 | 1.505 | 0.223 |
| Urban district | 55 | 11.63 | 57.95±15.37 | 1.505 | 0.223 |
| Primary caregiver |  |  |  |  |  |
| Spouse | 292 | 61.74 | 60.68±12.44 | 0.532 | 0.588 |
| Self | 3 | 0.63 | 53.33±20.82 | 0.532 | 0.588 |
| Children | 178 | 37.63 | 60.72±12.03 | 0.532 | 0.588 |
| Religious belief |  |  |  |  |  |
| Yes | 50 | 10.57 | 62.00±10.86 | 0.818 | 0.414 |
| No | 423 | 89.43 | 60.49±12.49 | 0.818 | 0.414 |
| Personality |  |  |  |  |  |
| Introverted | 144 | 30.44 | 60.32±11.98 | 0.080 | 0.923 |
| Extroverted | 160 | 33.83 | 60.73±12.65 | 0.080 | 0.923 |
| Intermediate | 169 | 35.73 | 60.86±12.37 | 0.080 | 0.923 |
| Type of work |  |  |  |  |  |
| Mental work | 188 | 39.74 | 61.32±12.08 | 1.032 | 0.357 |
| Physical labour | 111 | 23.47 | 61.19±12.16 | 1.032 | 0.357 |
| Mixed mental and physical work | 174 | 36.79 | 59.59±12.68 | 1.032 | 0.357 |
| Smoking |  |  |  |  |  |
| Yes | 255 | 53.91 | 61.17±11.74 | 0.995 | 0.320 |
| No | 218 | 46.09 | 60.04±12.97 | 0.995 | 0.320 |
| Alcohol use |  |  |  |  |  |
| Yes | 246 | 52.01 | 61.35±11.78 | 1.291 | 0.197 |
| No | 227 | 47.99 | 59.89±12.87 | 1.291 | 0.197 |
| Family history of stroke |  |  |  |  |  |
| Yes | 168 | 35.52 | 60.87±12.61 | 0.285 | 0.776 |
| No | 305 | 64.48 | 60.53±12.19 | 0.285 | 0.776 |
| Number of stroke episodes |  |  |  |  |  |
| Once | 384 | 81.18 | 60.80±12.19 | 0.553 | 0.581 |
| ≥2 times | 89 | 18.82 | 60.00±12.95 | 0.553 | 0.581 |
| Limb dysfunction |  |  |  |  |  |
| Left side | 269 | 56.87 | 60.12±12.53 | -1.079 | 0.281 |
| Right side | 204 | 43.13 | 61.35±12.05 | -1.079 | 0.281 |
| Number of chronic diseases |  |  |  |  |  |
| 1 | 243 | 51.37 | 61.23±12.14 | 1.539 | 0.216 |
| 2 | 195 | 41.23 | 60.53±12.41 | 1.539 | 0.216 |
| ≥3 | 35 | 7.40 | 57.34±12.93 | 1.539 | 0.216 |

Note. Values are n (%), mean ± SD, or health-behaviour score mean ± SD. *P≤0.05.
